# Psychiatric residents’ experience about Balint groups: A qualitative study using phenomenological approach in Iran

**DOI:** 10.30476/jamp.2020.85161.1164

**Published:** 2020-07

**Authors:** SEYYED TAHA YAHYAVI, MOZHGAN AMINI, FATEMEH SHEIKHMOONESI

**Affiliations:** 1 Department of Psychiatry, Roozbeh Hospital, School of Medicine, Tehran University of Medical Sciences, Tehran, Iran; 2 Psychiatry and Behavioral Sciences Research Center, Addiction Institute, Mazandaran University of Medical Sciences, Sari, Iran

**Keywords:** Psychoanalytic therapy, Psychiatry, Residency, Qualitative research

## Abstract

**Introduction:**

Previous research has shown that Balint group is effective in the professional and personal development of residents. The aim of this study was to investigate the experience of psychiatric residents about Balint groups.

**Methods:**

This is a qualitative study using a phenomenological approach. Seven sessions of the Balint groups were held with a number of psychiatric residents at Mazandaran University of Medical Sciences. Two of the leaders were psychoanalytic psychotherapist. Finally, eight in-depth semi-structured interviews and focused group interview were conducted. Data were collected by tape recorded interviews. Data were analyzed using MAXQDA-10 software.

**Results:**

Three main themes were obtained from the interviews that included "Early experiences", "Touching the Balint group" and "Relationship with Balint group". The category of “Early experiences” included three categories of "defenseless", "fire under the ashes" and "deep feeling". Touching the Balint group theme included categories such as "Empathetic", "I am not the only one ...", "Releasing", "Reading story", "This patient", and "Getting closer". The relationship with Balint group theme included three categories of "first of all", "attachment" and "courage and time".

**Conclusion:**

Based on the findings of this study, while some aspects of Balint group are stressful but ultimately improve the emotions and better understanding of the patient. This research shows incorporating Balint group into the educational program and curriculum of psychiatric residents in Iran might be helpful, but more qualitative and quantitative research is necessary.

## Introduction

Exposure to emotionally difficult situation puts the physicians at risk of burnout and compassion fatigue ( 1 , 2 ). Previous studies have shown decreased quality of life enjoyment and satisfaction in medical students compared to other non-medical students ( 3 , 4 ). Balint groups are the physicians which provide peer support and discussion about doctor-patient relationship. Participation in Balint group is associated with the reduced stress and burnout symptoms and increased job satisfaction in doctors and it might help the physicians handle a demanding work life. Balint groups can play a special role in medical education in general and in the training of residents and the psychiatric profession in particular ( 5 - 8 ).

Yazdankhahfard et al. (2019) studied the review articles on the use of the Balint groups in medical education. The findings of this research indicate that Balint groups can promote professional development in medical students and residents ( 9 ).

In a qualitative study by Simon Graham et al. (2009), the psychiatric residents and counselors who participated in Balint group were interviewed. The themes obtained were: “Understanding the clinical case dynamics”, “Developing awareness of own feelings from others”, “Introducing a new perspective or conceptual framework”, “Talking about feeling engendered by patients”, and “Ability to stay with feelings”. The authors suggested that according to the Bion’s theory the Balint groups may play as a "container" role for one's emotions ( 10 ). In the Marty Player et al.’s (2018) study, 18 family medicine residents from the University of Trident who attended the Balint group for 24 months were interviewed. Positive and negative themes were obtained from the interviews. Some positive themes were being the physician that the patient needs, empathy, reflection, blind spots, bonding, venting, perspectives, and some negative themes included repetition, uneasiness, and uncertain impact ( 11 ).

There are some studies about the effect of Balint group on improving communication skills in medical students ( 12 - 16 ), but there is not any research about this issue in Iran. The purpose of this study was to investigate the experience of psychiatric residents in Balint groups. This was a qualitative (phenomenology) study. The emphasis in phenomenology research is on the meaning of lived experience. We conducted this type of research to describe the residents’ experience. Using phenomenology study, we could understand the deeper meaning or significance of Balint group.

## Methods

This study was a qualitative research using a phenomenological approach. The research was done after a seven-session Balint group in Zare Hospital in the North of Iran in 2019. The Balint group was held every month for psychiatric residents and the group was closed. The group had two leaders; both of them were psychiatrists and psychoanalytic therapists who had completed the Balint leadership workshops. The role of the leaders in the group was protecting the framework and guiding the discussion with minimal intervention. The Balint group meetings were held for 60 minutes. 

The sample size used in qualitative research methods is often smaller than that used in quantitative research methods. An extremely large number of articles, book chapters, and books recommend guidance and suggest 5 to 50 participants as adequate ( 17 ). The inclusion criteria of the study were participating in the Balint group. Eight psychiatric residents volunteered to participate in the study after seven-session Balint group.

 The interviewer was a psychiatrist who had a background in conducting qualitative research and familiar with the Balint group process.

Individual interviews and focus groups are the most common methods of data collection used in qualitative health care research. Interviews can be used to explore the views, experiences, beliefs and motivations of individual participants. The focus group uses group dynamics to generate qualitative data ( 18 ). In this research, we use both methods for obtaining more accurate data.

Interviews were a one-hour focus group and 8 individual, in-depth semi-structured interviews that lasted about 30-40 minutes. Participants in the group and individual interviews were the same. After introducing him/herself, expressing the goals and obtaining permission to record conversations, the interviewer in the focus group started his/her interview with the open-ended question "What was your experience of attending the Balint group?" And the group process moved the discussion forward and the interviewer did only a few interventions to maintain the focus group framework. In individual interviews, the interviewer, after expressing his intentions and obtaining consent to record, opened the interview with the question "What was your personal experience of attending the Balint groups?" Then, with regard to the participants’ responses, developing and deepening questions were raised. 

Individual and focus group interviews were recorded, typed, and analyzed by using MAXQDA-10 software. Data were collected primarily through interviews. Open-ended questions were used. Interpretative phenomenological analysis (IPA) was done. IPA was the method of choice because it involves the qualitative analysis of a phenomenon based on the individuals’ experiences.

Analysis started by reading all the data repeatedly to achieve immersion and obtain a sense of the whole. Data were read word by word to extract the codes by first highlighting the exact words from the text that appear to capture the key thoughts or concepts. Analysis was done by encoding the statements and categorizing them by repeated readings by two experienced psychiatrists, one of whom was the interviewer; finally, the themes, categories and codes were classified. 

### Rigor

To evaluate the data accuracy, the researchers used credibility, dependability, confirmability and transferability criteria ( 19 ). In order to achieve the credibility of data, we performed member checking by the participants. Peer checking by the researchers was done to determine the confirmability of data. In order to determine the dependability of the data, an external observer from the research team, who was familiar with the Balint group was consulted. In order to achieve transferability, the participants were enrolled from different levels of residency according to maximum variation sampling.

### Ethical considerations

Participants entered the study voluntarily, after signing informed consent. They were told they could interrupt the interview at any time they wish, and if the interview was difficult for them, it would stop. Voice recording in the focus group and individual interviews were performed with the consent of the participants. The ethics committee of Mazandaran University of Medical Sciences approved this study (Ethical code: IR.MAZUMS.REC.1398.3479).

## Results

The characteristics of the participants are shown in Table 1.

**Table 1:** The characteristics of the participants

participant	Age	Sex	Marital status	Level (Resident of the year)
P1	31	Female	Single	1
P2	32	Female	Single	2
P3	34	Female	Married	2
P4	28	Female	Single	1
P5	35	Female	Married	1
P6	42	Female	Married	3
P7	42	Male	Married	1

Based on the results of the data analysis of the interviews with the focus group and individual interviews, three categories were identified that included "Early experiences", "Touching the Balint group" and "Relation with the Balint group". Each of these categories consisted of several subcategories and each subcategory consisted of several codes (Table 2). 

**Table 2:** Themes, categories and codes related to content analysis findings

Categories	Subcategories	Codes	Meaning units
Early experiences	Defenseless	I exposed myself	
		The words of others	(P3): "When I was expressing about my case, I felt that I was exposing myself." Another participant (P5) said: “It would be better if the people of the group was from the same level.” That's how you can trust better and empathy is better. It is better for everyone to choose their own group. As we had just one group there was practically no other option, but working with some group mates was difficult because of the history that we had."
		Is s/he staying here?	
		Fixed, familiar and limited people	(P6): "I think the most important role of a leader is to stop if anyone was to judge or analyze”.
		Supportive leader	
	Fire Under Ash	There is a lot of talk and feeling behind it	(P2): "At the Balint meetings I realized that there was a lot of talk and feeling behind it."
		I couldn't even tell myself	
	Deep Feeling	In my heart	(P5): "My heart was squeezed "When I started talking and telling my story, my heart was squeezed. All the emotions that I had not thought of them for a long time were there." (P1) said: "After the Balint group was over, as if the storm has subsided. Everything has calmed down. A pleasant calm!"
		Relaxation after the storm	
Touching the Balint group	Empathetic	It was real to me	(P7): "All the consolation I was given was real to me".
		If we were him/her	(P6) said: "We could imagine what would have happened to us, if it were for us".
	I'm not the only one	Others	(P3) said: "How similar is all experiences when they are facing with their patients”.
		Calm me down	(P1) said, “Common Experience and common Sense makes one to be calm”.
	To be released	Become light	(P4): “After telling about the case I was released."
		Node	(P1): "It makes it easy to talk about the case and the extra burden I felt it was taken away from my shoulder.
		Digestion	(P7): "It made it easier for me to digest it after presenting it in the Balint Group."
	Reading the story	Experience with hearing	(P6): "I could experience all the feelings that they say." (P4): "It is like reading a story or a book. One gets emotional about the characters in the story and the whole story affects him for a while".
		Story, narration	
	This patient	New aspects	(P5): "I understood why this special case was so carved in my mind."
		To become sensitive	
	Getting closer	I	(P5): "A new meaning about death has been formed in me ".
		Life	(P7): "I realized that there are things in our lives that will never be resolved."
Relation with Balint group	At the Beginning	Worry	(P1): “The first session I didn't trust to talk about my emotion"
		Confusion	
		Curiosity	(P1): "I had ambivalency in the first session, and I was worried about the group”.
	Attachment	I liked him/her/it	(P3) stated: “After 5 or 6 sessions I felt how much I love this group."
	Courage and time	It takes time	(P7) said: “Balint group has increased my courage to be able to comment on others".
		Be seen	(P2): "In Balint group you dare to say and be seen."

### 1-Early experiences

The main theme of "Early experiences" refers to people's experience of early exposure to the Balint group.

Defenseless

 One of the related codes was “I exposed myself”. The code for "The words of others" referred to the participant's fear and concern about the harassment of words of other participants in the group. The other code was "Fixed, Familiar and Limited people". One participant (P5) said, *“It would be better if the individuals in the group were from the same level.”* The next code was "Leader, Supporter". It pointed to the role of the Balint group leaders in creating a sense of trust and producing the proper atmosphere. 

1.2. Fire Under Ash

The second subcategory was "Fire under Ash". This category itself consisted of two codes. The code was "a lot of talk and feeling behind it". It referred to defining the cases in the Balint group and expressing feelings and emotions on behalf of the group invoked old or suppressed memories of others, and led the things from the subconscious to the conscious. One participant (P1) used the metaphor of "fire under the ashes". 

1.3. Deep Feeling

### 2. Touching the Balint group

2.1. Empathy

This subcategory consisted of two codes. Both codes indicated empathy. 

2.2. I'm not the only one...

This subcategory referred to creating a sense of unity between the members and achieving peace after this feeling. One participant (P3), said, *“How similar are all experiences when they face with their patients”*.

2.3. To be released

This subcategory consisted of three codes and had the concept of emotional and cognitive relief. These included "lightening", "node" and "digestion". 

One participant (P3) said: *“After telling about the patient, it became easier for me to cope with the case”*.

2.4. Reading the story

The "narrative" code referred to the Balint group experience as a narrative. Another code in this category was the "Experience with hearing". This code explained that the "narrative" which was expressed in the Balint group created an emotional effect as much as an experience in the participants. 

2.5. This patient

This subcategory referred to the exploration of the particular aspects that each case had. "This patient" was in the mind of the presenter for some reason that was revealed in the Balint group and now the person himself or herself is aware of it. 

2.6. Getting closer

This subcategory explains the impact of the Balint on changing people's attitudes to "life" and themselves. One participant (P5) said: *"A new meaning about death has been formed in me "*.

### 3. Relationship with Balint group

This category referred to the feelings that was formed in individuals in relation to the Balint group during meetings. As if the Balint was an "object" and took time to form a relationship.

3.1. At the Beginning

This subcategory referred to the feelings that people developed when they started the Balint group. Participant one (P1) said: *“In the first session, I didn't trust to talk about my emotion”*. 

3.2. Attachment

One participant (P1) said,* "I was absent in one session and I felt I was missing something"*. 

3.3. Courage and time

This subcategory indicated that over time and by attending the sessions, the trust was formed and the courage of individuals to express their feelings and their views was enhanced, but this is something that needs time. One participant (P7) said, *“Balint group has increased my courage to be able to comment on others”.*

## Discussion

Based on the analysis of data, the experience of psychiatric residents in participating in Balint groups includes the feeling of defenseless which refers to the fear of expressing emotions and thoughts in public and the fear of being judged and harmed, facing with deep dynamics and dynamics that are not aware of them, and they are like the fire beneath the ashes and it can sparkle at any moment. In fact, participants find themselves protected from the fire under their ashes. At this point, people are faced with deep feelings and experience powerful emotions that are felt in their heart, which first intensifies and then relaxes. The individual must release and let himself float. He must trust in the leader and other members of the group and expose himself. In this plunge, it is as if emotions were peaked, and hearts were squeezed; the words and emotions that were buried and outdated were re-experienced.

As if, emotions are raising and the hearts are squeezed and the old and buried words and emotions that are re-experienced in this floating. The beginning of sinking is dipping and is accompanied by the intense emotions, and then it comes to rest. People in the Balint group found the space empathically. For the clinical case presenter, the feeling of being understood is created.

Comparison of the findings of this study with the results of Marty Player’s research (2018), the themes of reflection, empathy, blind spots, originality, attachment, acceptance, perspective, and personal experiences were the themes also addressed in the present study. However, due to the long duration of that research, the negative themes were obtained that were not found in this study. The medical context that the patient needed was not also included in this study ( 11 ).

The codes of "fixed and familiar people" and "supportive leader" and then "curiosity" had the highest frequency in the interviews, respectively, so that practically the code of familiar and limited persons was obtained more than once in many interviews. All three of these codes are related to the small number of Balint groups and not fixing of the dynamics of the group. There may be usually a curiosity at the beginning of any new work that will be resolved over time and replaced with reflection. It is understandable that people are concerned about the framework of the group and need to have a predictable framework in which the group leader has a supportive role. The reason for the drop out in the Balint group over time was that none of the participants had a history of attending the group therapy. Having conflicts with other participants in the Balint group, especially in the first sessions, due to the presence of psychiatric residents from different levels, is predictable and a lot of code repetition can reflect it.

The limitations of this study were the small number of participants and Balint group sessions, which was inevitable due to resource constraints. The authors did not find a valid questionnaire to evaluate the quality of the Balint group.

## Conclusion

Based on the findings of this study, while some aspects of Balint group are stressful, it ultimately improves the emotions and better understanding of the patient. Using this study and similar research can be helpful for providing a valid and reliable tool for evaluating the quality of Balint groups. With current evidence, it may still be too early to include the Balint groups in educational curriculum of psychiatry residents in Iran, but quantitative and qualitative research in this regard can provide the context for this action. The authors suggest that psychiatry residents may be more familiar with group therapy and it is easier for them to deal with the Balint group because of their field of study requirements; however, as it is the experience of many countries, maybe moving toward creating Balint groups in other disciplines is inevitable. It can be accomplished through research on Balint group and its impact on general practitioners and other specialized disciplines. 
